# Environmental exposures to organophosphorus flame retardants in early pregnancy and risks of gestational diabetes mellitus: a nested case–control study

**DOI:** 10.1038/s41598-024-64557-9

**Published:** 2024-06-14

**Authors:** Qi Lang, Jiali Sun, Xiangyuan Yu, Shudan Wei, Jinyan Wei, Min Zhang, Chaochao Zhao, Jun Zhang, Dingyuan Zeng, Bo Huang

**Affiliations:** 1 Clinical Laboratory Center, First Affiliated Hospital of Guilin Medical University, 109 Ring City North Second Road, Guilin, 541004 Guangxi China; 2 Guangxi Key Laboratory of Environmental Exposomics and Entire LifeCourse Health, Guangxi Health Commission Key Laboratory of Entire LifeCourse Health and Care, School of Public Health, Guilin Medical University, 1 Zhiyuan Road, Guilin, 541199 Guangxi China; 3 Ministry of Education and Shanghai Key Laboratory of Children’s Environmental Health, Xin Hua Hospital, Shanghai JiaoTong University School of Medicine, 1665 Kongjiang Road, Shanghai, 200092 China; 4 Guangxi Health Commission Key Laboratory of Birth Cohort Study in Pregnant Women With Advanced Age, Liuzhou Maternity and Child Healthcare Hospital, 50 Yingshan Street, Liuzhou, 545001 Guangxi China

**Keywords:** Organophosphorus flame retardants, Gestational diabetes mellitus, Weighted quantile regression, Bayesian kernel machine regression, Nested case–control study, Endocrinology, Health care, Risk factors

## Abstract

OPFRs are emerging environmental pollutants with reproductive and endocrine toxicity. This study aimed to examine the association between environmental exposure to OPFRs during early pregnancy and GDM. This nested case–control study was based on a birth cohort that was constructed at a maternal and child health hospital, including 74 cases of GDM among 512 pregnant women. The OPFRs, including TBP, TBEP, TCEP, TDCPP, TMCP, TOCP, and TPHP during 10–14 weeks of pregnancy were determined using GC–MS. The association between the OPFRs and GDM was assessed using WQS and BKMR models. The levels of OPFRs were significantly elevated in GDM patients (60) compared with the controls (90). The WQS analysis showed that mixtures of the OPFRs were significantly associated with GDM (OR 1.370, 95% CI 1.036–1.810, *P* = 0.027), and TBP, TPHP, and TMCP were the major contributors to the mixed exposure effect. In the BKMR model, individual exposure to TBP, TPHP, and TMCP, and the interaction of TMCP with TBP and TPHP were significantly associated with GDM. Environmental exposure to OPFRs is positively associated with GDM. These findings provide evidence for the adverse effects of OPFR exposure on the health of pregnant women.

## Introduction

Organophosphorus flame retardants (OPFRs) are a class of compounds with similar structures, most of which have a phosphate triester structure^1^. As a substitute for polybrominated diphenyl ethers (PBDEs), the production and usage of OPFRs have dramatically increased worldwide since 2009, and PBDEs have been listed as persistent organic pollutants in the Stockholm Convention^1^. OPFRs are widely used in various industrial products and daily necessities such as polyurethane foam, building materials, furniture, electronic appliances, and textiles^2–7^. Because OPFRs are added to polymer materials through physical incorporation rather than chemical bonding with the product matrix, they are easily released into the environment via volatilization, dissolution, leaching, and abrasion. OPFRs have been detected in various environmental media and bio-samples, such as indoor air and dust, drinking water and soil, human hair, nails, breast milk, urine, blood, decidua of pregnant women, and embryo villi^8–11^. Moreover, OPFRs have shown carcinogenicity, neurotoxicity, liver toxicity, and reproductive and developmental toxicity^12–18^. However, data on the association between OPFR exposure and pregnancy complications are rare.

Gestational diabetes mellitus (GDM) refers to impaired glucose tolerance that occurs or is first discovered during pregnancy, and is the most common pregnancy complication, with incidences of 1–14% worldwide^19^. GDM may be associated with various adverse pregnancy and birth outcomes, such as gestational hypertension, preeclampsia, polyhydramnios, postpartum hemorrhage, macrosomia, hypocalcemia, respiratory distress syndrome, and polycythemia^20^. Affected mothers and children are more susceptible to cardiovascular diseases, type 2 diabetes mellitus (T2DM), and hypertension later in their life^20–24^. The etiology of GDM remains unclear, but GDM is known to be associated with a history of GDM, low physical activity, advanced maternal age, overweight or obesity, and exposure to environmental hazards, such as heavy metals and persistent organic pollutants^25^.

The endocrine-disrupting effects of OPFRs prompted us to explore the association between OPFRs and GDM. In this study, urinary tributyl phosphate (TBP), tris (2-butoxyethyl) phosphate (TBEP), tris (2-chloroethyl) phosphate (TCEP), tris (1,3-dichloro-2-propyl) phosphate (TDCPP), tri-m-cresyl phosphate (TMCP), tri-ortho-cresyl phosphate (TOCP), and triphenyl phosphate (TPHP) during early pregnancy were determined based on a nested case–control study. These OPFRs are the major species exposed in the Chinese population^26,27^. This study aimed to examine the association between environmental exposure to OPFRs and GDM, using weighted quantile regression (WQS) and Bayesian kernel machine regression (BKMR) analyses.

## Materials and methods

### Study populations

A birth cohort (2042 mother–child pairs) was developed in Liuzhou Maternity and Child Healthcare Hospital for women and children health research with follow-up via linkage to the medical records of the hospital from September 2016 to December 2018. This study was nested in the cohort study that included 74 GDM cases among 512 pregnant women from August to December 2018. The diagnostic criteria for GDM were fasting blood glucose ≥ 5.1 mmol/L, one-hour blood glucose ≥ 10.0 mmol/L, or two-hour blood glucose ≥ 8.5 mmol/L in the oral glucose tolerance test (OGTT) performed in 24–28 weeks of pregnancy^28^. Women with hypertension, diabetes before pregnancy, thyroid diseases, liver and kidney diseases, infectious diseases, GDM with any other pregnancy complications, communication barriers, or loss to follow-up were excluded. Ultimately, 14 GDM cases were excluded, and 60 GDM cases were recruited and matched with 90 controls (cases:controls = 1:1.5). All participants met the following inclusion criteria: (1) permanent residents of Liuzhou City and aged 20–45; (2) gestational age between 10 and 14 weeks as enrolled; (3) complete questionnaires; (4) available plasma and urinary samples; and (5) singleton pregnancy. Information, including maternal age, race, occupation, education level, household income, smoking history, number of pregnancies, history of diabetes and GDM, and family history of diabetes, was collected through face-to-face interviews to complete the questionnaire. In addition, the pregnant women’s blood pressure, height, and weight were measured and recorded, and blood and urine samples were collected during the first antenatal examination in the first trimester. Maternal body mass index (BMI) was calculated by dividing weight (kg) by the square of height (m^2^). Pregnancy complication information was obtained from the hospital medical record system. All participants signed an informed consent form, and the study was approved by the Ethics Committee of Guilin Medical University (No. GLMC20131205). We ensured that the study protocols had coincided with relevant guidelines and regulations, including the ethical principles for medical research involving human subjects declared by the World Medical Association (Helsinki).

### Determination of urinary organophosphorus flame retardants

OPFRs in urine were determined at the Changchun Institute of Applied Chemistry, Chinese Academy of Sciences, using a high-performance gas chromatography-tandem triple quadrupole mass spectrometer (GC–MS) (Agilent 7000D, USA). Standard reagents of the seven OPFRs, including TBP (CAS: 126-73-8, purity 98.00%), TBEP (CAS: 78-51-3, purity 95.00%), TCEP (CAS: 115-96-8, purity 98.00%), TDCPP (CAS: 13674-87-8, purity 96.00%), TMCP (CAS: 13674-84-5, purity 98.00%), TOCP (CAS: 78-30-8, purity 98.00%), and TPHP (CAS: 513-08-6, purity 98.00%) were purchased from the China National Standards Center. 100.0 mg of the standard reagents TBP, TBEP, TCEP, TDCPP, TMCP, TOCP, and TPHP were dissolved in anhydrous ether (10 mL), and 100 μL of the solution was placed in a conical test tube, dried with nitrogen at room temperature, and then re-dissolved in 10 mL of *n*-hexane, which was diluted with n-hexane into series at 0, 1, 2, 5, 10, 20, 50, 100, and 200 ng/mL to construct standard curves. Morning urine samples were collected during the first trimester of pregnancy (10–14 weeks) and stored at − 80 °C. Before analysis, urine samples were thawed at 4 °C and purified using a SampliQ OPT (3 mL, 60 mg) solid-phase extraction column by activation with 3 mL methanol for 30 min. Then 1.68 g of Na_2_SO_4_ was added to the column, and 1 mL of the urine sample was loaded and balanced for 5 min and drained at a rate of 10–20 drops/min. After 10 mL of *n*-hexane was added to remove non-polar impurities in the urine at a rate of 10–20 drops/min and drain, the column was eluted with 6 mL of ether-*n*-hexane (9:1, v/v). The eluent was collected and dried with nitrogen and then redissolved in 10 mL of n-hexane for GC/MS analysis with a 122-3832E capillary column (30 m × 0.25 mm × 0.25 μm, Agilent). The carrier gas was high-purity helium (purity ≥ 99.999%) and the flow rate was 2.25 mL/min. The initial temperature of the column was 80 °C (held for 1.35 min), which was then increased to 280 °C (held for 13 min) at 12 °C/min. The injection volume was 1.0 μL without a shunt. An electron bombardment (EI) ionization source was used for mass analysis with an ionization voltage of 70 eV, an ion source temperature of 230 °C, and a mass spectrum quadrupole temperature of 150 °C. A selected ion monitoring (SIM) model was used for analysis. The SIM masses and retention times were as follows: TBP (155/99, 8.00 min), TBEP (299/125, 17.60 min), TCEP (251/249, 13.20 min), TDCPP (380/191, 18.10 min), TMCP (277/125, 13.20 min), TOCP (367/165, 21.50 min), and TPHP (325/170, 19.20 min). The OPFRs were quantified according to the linear regression equations of the standard curves, with the concentration (x) of each OPFR corresponding to the peak area (y). The linear correlation coefficients (squares) for all OPFRs were greater than 0.997. The limits of quantitation (LOQ) were calculated with a signal-to-noise ratio (S/N) of 10/1, and the LOQs for TBP, TBEP, TCEP, TDCPP, TMCP, TOCP, and TPHP were 0.342, 0.627, 0.423, 0.933, 0.387, 0.586, and 0.298 ng/mL, respectively. For the recovery study, eighteen randomly selected urinary samples (six for each spiked concentration) that had been previously checked were spiked with the standard compounds of the seven OPFRs at three concentrations (1, 5, and 20 ng/mL) that covered most of the sample analyses. The samples were then processed and assayed as previously described, and the recovery rates and relative standard deviation (RSD) of these fortified samples were calculated, yielding 85–115% of recovery rates and 2.3–10.2% of RSD in this study. Creatinine levels in the urine samples were measured using the creatine oxidase method (Nanjing Jiancheng, Cat. No.C011-2-1), and the OPFRs concentrations were corrected with creatinine.

### Statistical analysis

Statistical analyses were conducted using R 4.2.1. Continuous data with normal distribution were presented as mean ± standard deviation (x̄ ± SD) and tested using Student’s t-test. Categorical data were described by frequencies (n (%)) and tested using the chi-square test. A log10 transformation was applied for the levels of OPFRs to address skewed data presented as median (interquartile range). Spearman's correlation analysis was used to examine the correlation between spices of the OPFRs in control populations.

The overall effect of mixed exposure to OPFRs on GDM and the weighted quantile sum (WQS) indices of discrete OPFRs contributing to GDM were evaluated using the WQS analysis. In the analysis, 30% of the data was used as the test dataset, 40% for validation, and 30% for prediction. The β1 coefficient was set as positive or negative, and 10,000 iterations were performed to explore the positive or negative correlation between the mixed exposure of OPFRs and GDM^29^.

To overcome the influence of collinearity and explore the potential interaction of OPFRs, a Bayesian kernel machine regression (BKMR) model was used to assess the association between exposure to OPFRs and GDM^30^. The following questions were addressed: (1) The overall effect of the OPFRs on GDM using their median exposure levels as a reference; (2) the effect of individual OPFRs on GDM, in which the potential sequential outcome (GDM) at the 25th to 75th percentiles of a single OPFR was calculated while the other OPFRs were fixed at their 25th, 50th, and 75th percentiles, respectively; (3) the relationship between an OPFR and GDM using the univariate exposure–response function when other OPFRs were at their median levels; (4) the interaction between OPFRs on GDM by determining the exposure–response relationship of one OPFR combined with another OPFR at the 25th, 50th, and 75th percentiles, respectively; and the other OPFRs were fixed at their median exposure levels. Because there was a high correlation between the OPFRs, we used the probit function in the Markov chain Monte Carlo (MCMC) algorithm to implement probit regression. After 10,000 iterations of hierarchical variable selection, TBP, TBEP, TCEP, TDCPP, and TOCP were classified as Group 1, and TMCP and TPHP were divided into Group 2. The BKMR formula was Yι^*^ = h (Group1 = [TBP, TBEP, TCEP, TDCPP, TOCP], Group2 = [TMCP, TPHP]) + ®*x*ι + ∑ι. Yι^*^ was the binary variable (1 = GDM, 0 = control). h () is the exposure–response function of the exposure and outcome, and *x*ι, ®, and ∑ι are the covariate, coefficient, and residual terms, respectively. The group posterior inclusion probability (groupPIP) was estimated. The conditional posterior inclusion probability (condPIP) was computed, representing the probability that a particular OPFR within a group was included in the model. A PIP threshold of 0.50 is usually used to determine if it is important^31^.

### Ethics statement

The studies involving human participants were reviewed and approved by the Medical Ethics Committees of Guilin Medical University (No. GLMC20131205). The patients/participants provided written informed consent to participate in this study.

## Results

### Characteristics of study populations

The demographic characteristics of the study population (60 GDM patients and 90 controls) are shown in Table 1. There were no significant differences between the two groups in terms of BMI, SBP, DBP, ethnicity, occupation, smoking history, number of pregnancies, education level, household income, history of diabetes, or family history of diabetes (*P* > 0.05). However, maternal age and rate of GDM history were higher in the GDM group than in the control group (*P* < 0.05, *P* < 0.01) (Table 1).

**Table 1:** Characteristics of study populations.

Characteristics	Total population (n = 150) Mean ± SD or No (%)	Control (n = 90) Mean ± SD or No (%)	GDM (n = 60) Mean ± SD or No (%)	*P*-value
Maternal age (year)	32.234 ± 4.902	31.361 ± 4.836	33.543 ± 4.745	**0.007**
BMI (kg/m^2^)	21.624 ± 2.954	21.353 ± 2.601	22.031 ± 3.399	0.169
SBP (mmHg)	107.116 ± 9.730	105.995 ± 9.341	108.798 ± 10.134	0.084
DBP (mmHg)	69.829 ± 8.212	69.444 ± 7.734	70.405 ± 8.917	0.484
Nationality				0.227
Han	91 (60.667)	52 (57.778)	39 (65)	
Zhuang	46 (30.667)	27 (30)	19 (31.667)	
Others	13 (8.667)	11 (12.222)	2 (3.333)	
Education level				0.501
High school and below	53 (35.333)	32 (35.556)	21 (35)	
University and below	77 (51.333)	49 (54.444)	28 (46.667)	
Above university	20 (13.333)	9 (10)	11 (18.333)	
Household income (yuan)				0.432
0-	70 (46.667)	44 (48.889)	26 (43.333)	
50,000-	40 (26.667)	22 (24.444)	18 (30)	
100,000-	40 (26.667)	24 (26.667)	16 (26.667)	
Occupation				0.485
Office clerk	116 (77.333)	68 (75.556)	48 (80)	
Industrial worker	5 (3.333)	3 (3.333)	2 (3.333)	
Agricultural worker	4 (2.667)	2 (2.222)	2 (3.333)	
Others	25 (16.667)	17 (18.889)	8 (13.333)	
Smoking history				0.812
No	147 (98)	88 (97.778)	59 (98.333)	
Yes	3 (2)	2 (2.222)	1 (1.667)	
Number of pregnancies				0.186
Primipara	138 (96.667)	85 (94.444)	53 (88.333)	
Multipara	12 (3.333)	5 (5.556)	7 (11.667)	
History of diabetes				0.082
No	148 (98.667)	90 (100)	58 (96.667)	
Yes	2 (1.333)	0 (0)	2 (3.333)	
History of gestational diabetes				**0.028**
No	144 (96)	89 (98.889)	55 (91.667)	
Yes	6 (4)	1 (1.111)	5 (8.333)	
Family history of diabetes				0.529
No	143 (95.333)	85 (94.444)	58 (96.667)	
Yes	7 (4.667)	5 (5.556)	2 (3.333)	

*P-*values were estimated using Student’s t-test (log10-transformed data) or the chi-square test (frequencies). Bold numbers represent statistical significance (*P* < 0.05). *BMI* body mass index, *DBP* diastolic blood pressure, *SBP* systolic blood pressure.

### Levels of urinary OPFRs in GDM cases and their controls

The detection rate of all the OPFRs was greater than 90.0%. In the control group, the detection rates of TBP, TBEP, TCEP, TDCPP, TMCP, TOCP and TPHP were 98.89%, 98.89%, 98.89%, 97.78%, 98.89%, 98.89%, and 92.22%, respectively. In the GDM group, the detection rates for TBP, TBEP, TCEP, TDCPP, TMCP, TOCP, and TPHP were 98.33%, 100%, 100%, 98.33%, 98.33%, 96.67%, and 90%, respectively. There was no difference in the detection rates between the two groups. TDCPP was the most abundant OPFR in the study populations, followed by TBEP, TMCP, TOCP, TCEP, TPHP, and TBP. TDCPP and TBEP accounted for 74.90% of the total OPFR concentrations. The OPFRs levels in women with GDM were significantly higher than those in the control group (*P* < 0.01) (Table 2).

**Table 2:** Comparison of OPFR levels between GDM patients and their controls.

OPFRs	Detection rate (%)	Control (n = 90) (ng/mg creatinine)		GDM (n = 60) (ng/mg creatinine)		*P*-value
		*P*_50_	*P*_25_–*P*_75_	*P*_50_	*P*_25_–*P*_75_	
TBEP	99.33	14.770	5.194–45.856	31.074	12.918–87.763	**0.001**
TBP	98.67	1.019	0.717–1.894	2.786	1.587–6.810	**< 0.001**
TCEP	99.33	1.465	0.911–3.008	3.122	1.560–6.649	**< 0.001**
TDCPP	98.00	14.066	7.247–35.718	51.389	22.098–143.938	**< 0.001**
TMCP	98.67	3.997	1.799–8.005	4.228	2.783–12.575	**< 0.001**
TOCP	98.00	1.872	1.056–3.607	4.790	1.962–11.057	**< 0.001**
TPHP	91.33	1.292	0.828–3.054	2.659	1.199–6.110	**< 0.001**
Total	97.62	2.900	1.091–8.527	5.907	2.208–22.883	**< 0.001**

OPFR levels were presented as medians (interquartile ranges). *P*-values were derived using the Student’s t-test (log10-transformed data). Bold numbers represented statistical significance (*P* < 0.05). *GDM* gestational diabetes mellitus, *OPFR* Organic phosphate flame retardant, *TBP* tributyl phosphate, *TBEP* tris (2-butoxyethyl) phosphate, *TCEP* tris (2-chloroethyl) phosphate, *TDCPP* tris (1,3-dichloro-2-propyl) phosphate, *TMCP* tri-m-cresyl phosphate, *TOCP* tri-ortho-cresyl phosphate, *TPHP* triphenyl phosphate.

### Correlation between OPFRs in control group

**Figure 1:**
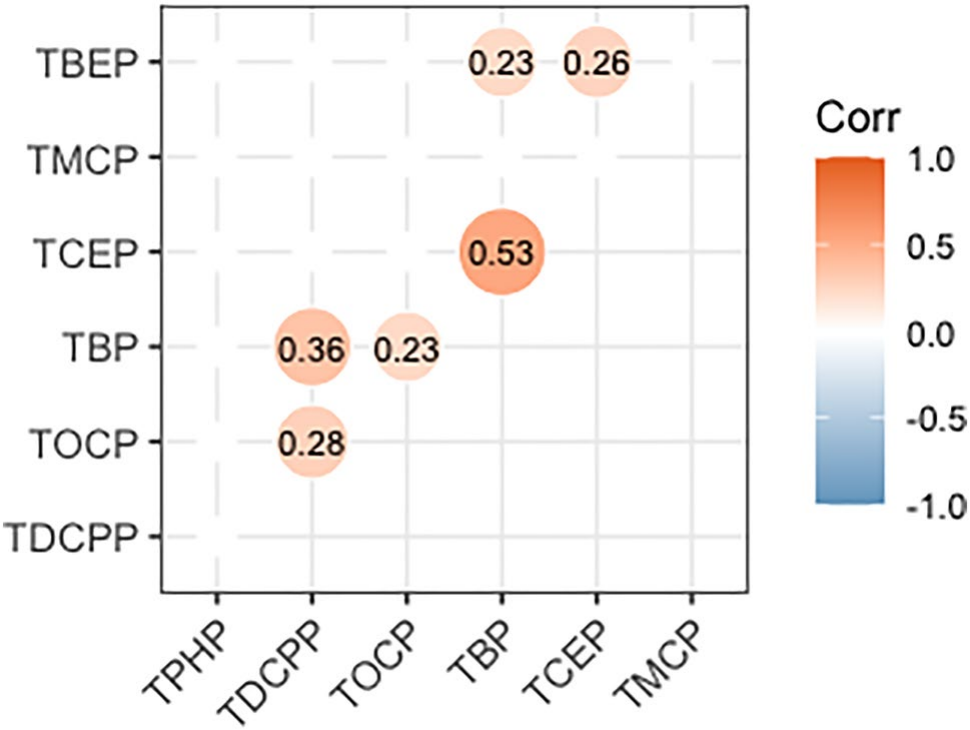
Heat map of the correlation between species of OPFRs in control populations. Spearman’s correlation coefficients (r) are shown in the cells. Correlations without statistical significance are hidden in the figure. *OPFRs* Organic phosphate flame retardants, *TBP* tributyl phosphate, *TBEP* Tris (2-butoxyethyl) phosphate, *TCEP* tris (2-chloroethyl) phosphate, *TDCPP* tris (1,3-dichloro-2-propyl) phosphate, *TMCP* tri-m-cresyl phosphate, *TOCP* tri-ortho-cresyl phosphate, *TPHP* triphenyl phosphate.

Correlation analysis revealed that TBP was strongly and positively correlated with TCEP. In addition, there was a positive correlation between TBP and TDCPP, TOCP, and TBEP, whereas TCEP was correlated with TBEP (Fig. 1).

### WQS index weights of OPFRs for their associations with GDM

**Figure 2:**
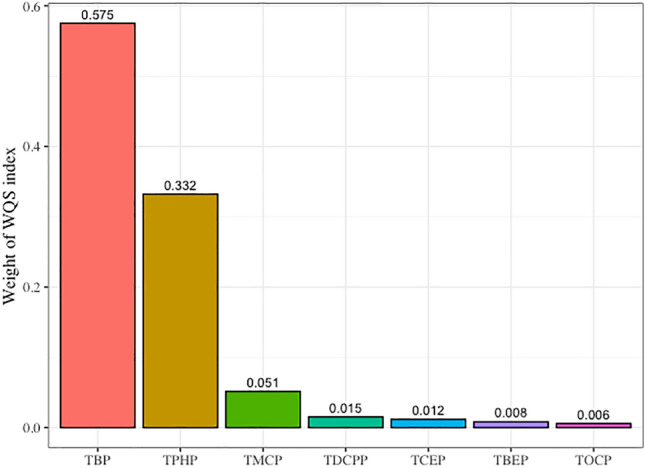
WQS index weight of OPFRs associated with GDM. The analysis was based on WQS regression modeled in a positive direction with respect to the outcome (adjusted for covariates of maternal age and GDM history). *GDM* gestational diabetes mellitus, *OPFRs* Organic phosphate flame retardants, *TBP* tributyl phosphate, *TBEP* Tris (2-butoxyethyl) phosphate, *TCEP* tris (2-chloroethyl) phosphate, *TDCPP* tris (1,3-dichloro-2-propyl) phosphate, *TMCP* tri-m-cresyl phosphate, *TOCP* tri-ortho-cresyl phosphate, *TPHP* triphenyl phosphate, *WQS* Weighted quantile sum.

After adjusting for maternal age and history of GDM, the WQS indices of OPFR mixtures were significantly positively associated with GDM (OR 1.370, 95% CI 1.036–1.810, *P* = 0.027) in the WQS regression model. TBP had the highest WQS index weight, accounting for 57.5% of the overall effect on the association with GDM, followed by TPHP, with a weight of 33.2% (Fig. 2). No significant association between the β1 coefficient and GDM was observed in the negative direction of analysis.

### Effect of exposure to OPFRs on GDM in BKMR analysis

**Figure 3:**
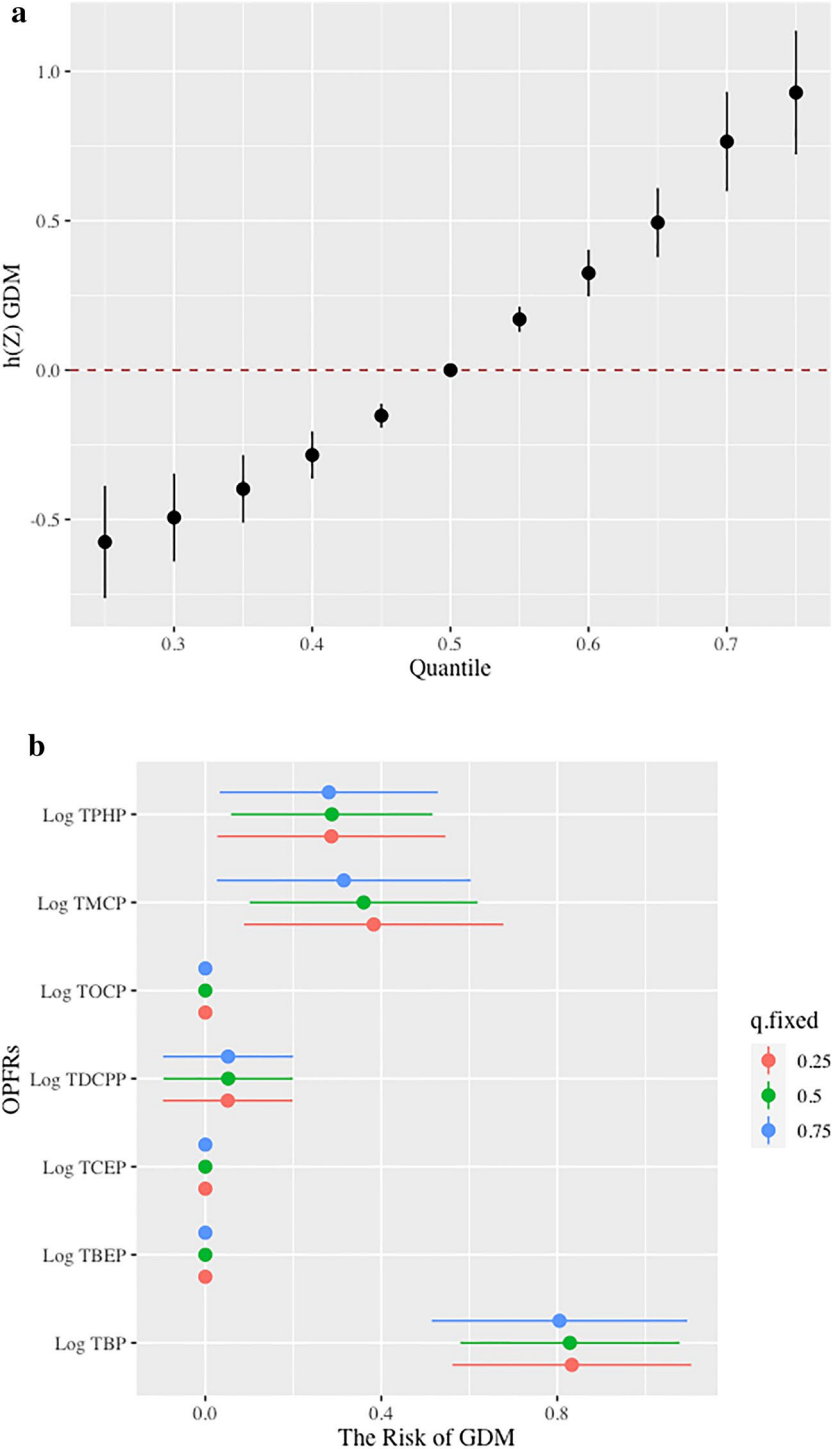

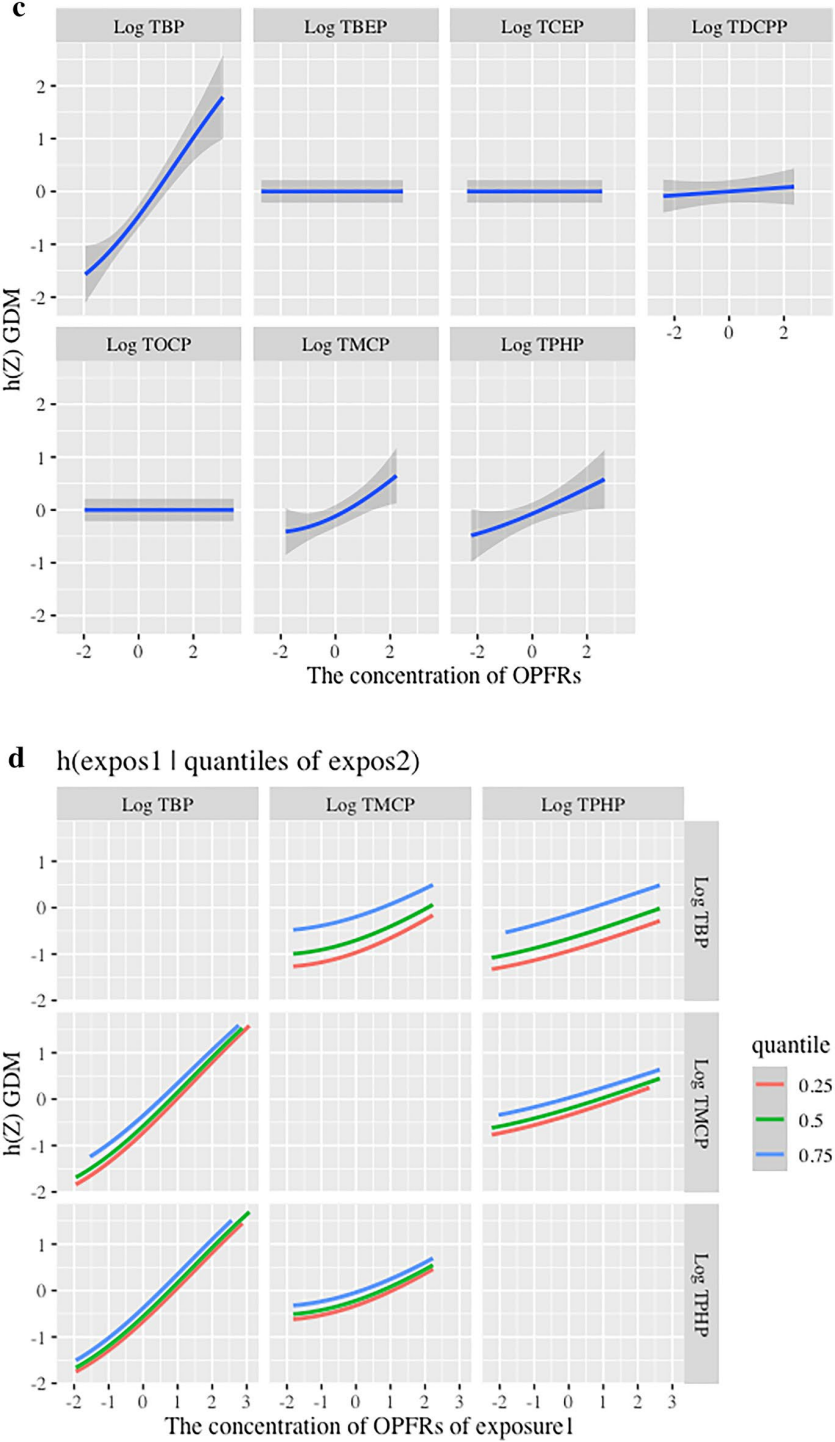
Association of exposure to OPFRs with GDM in BKMR analysis. (**a**) The overall effect of mixed exposure to OPFRs on GDM, while the exposure levels were higher than the median levels. (**b**) Association between individual OPFRs and GDM when the other OPFRs were at the 25th, 50th, and 75th percentiles. (**c**) The univariate exposure–response effect of individual OPFRs on GDM, while the other OPFRs were fixed at their median exposure levels. (**d**) The bivariate exposure–response function for one OPFR with another OPFR set at the 25th, 50th, and 75th percentiles, and the other OPFRs were fixed at their median exposure levels. Analyses were adjusted for maternal age and GDM history. *BKMR* Bayesian kernel machine regression, *condPIP* conditional posterior inclusion probability, *GDM* gestational diabetes mellitus, *OPFRs* Organic phosphate flame retardants, *PIP* posterior inclusion probability, *TBP* tributyl phosphate, *TBEP* Tris (2-butoxyethyl) phosphate, *TCEP* tris (2-chloroethyl) phosphate, *TDCPP* tris (1,3-dichloro-2-propyl) phosphate, *TMCP* tri-m-cresyl phosphate, *TOCP* tri-ortho-cresyl phosphate, *TPHP* triphenyl phosphate.

The PIPs derived from the BKMR model for the groups (Group PIP) and individual OPFRs (CondPIP) were listed in Table 3, showing that TBP, TPHP, and TMCP were important (CondPIP > 0.50). The overall association between OPFRs exposure and potential sequential outcomes was identified as a significant increase in GDM (Fig. 3a). TBP, TPHP, and TMCP were positively correlated with GDM, while their exposure levels ranged from the 25th to 75th percentiles (Fig. 3b). When the other OPFRs were fixed at the 50th percentile of exposure levels, TBP, TPHP, and TMCP were positively associated with GDM (Fig. 3c). Although TBP and TMCP were at different quantiles of exposure levels, the influence of exposure on GDM differed, suggesting an interaction between TBP and TMCP. A similar interaction was observed between TPHP and TMCP (Fig. 3d).

**Table 3:** PIPs of OPFR inclusion into GDM in BKMR analysis.

OPFRs	Groups	GroupPIP	CondPIP
Log TBP	1	1.000	**0.991**
Log TBEP	1	1.000	0.000
Log TCEP	1	1.000	0.000
Log TDCPP	1	1.000	0.009
Log TOCP	1	1.000	0.000
Log TMCP	2	0.937	**0.614**
Log TPHP	2	0.937	**0.586**

The analysis was adjusted for maternal age and GDM history. The bold numbers. represented “important” as PIP > 0.50. *BKMR* Bayesian kernel machine regression, *condPIP* conditional posterior inclusion probability, *GDM* gestational diabetes mellitus, *OPFR* Organic phosphate flame retardant, *PIP* posterior inclusion probability, *TBP* tributyl phosphate, *TBEP* Tris (2-butoxyethyl) phosphate, *TCEP* tris (2-chloroethyl) phosphate, *TDCPP* tris (1,3-dichloro-2-propyl) phosphate, *TMCP* tri-m-cresyl phosphate, *TOCP* tri-ortho-cresyl phosphate, *TPHP* triphenyl phosphate.

## Discussion

OPFRs have attracted increasing attention as emerging environmental pollutants owing to their adverse effects, particularly on maternal and child health. Based on a nested case–control study, we found that the levels of urinary TBP, TBEP, TCEP, TDCPP, TMCP, TOCP, and TPHP during early pregnancy were significantly higher in GDM patients than in the controls. A mixture of the OPFRs, individual exposure to TBP, TPHP, and TMCP, and interactions of TMCP with TBP and TPHP were significantly positively associated with GDM. TBP had the highest WQS index weight for GDM, followed by TPHP and TMCP, accounting for > 95% of the total weight. These findings provide evidence for the adverse effects of OPFR exposure on the health of pregnant women.

### Exposures of OPFRs in the population

Although certain PBDEs used as flame retardants have been banned or restricted in commercial mixtures, the production and usage of OPFRs has dramatically increased in recent years. In 1992, only 100,000 tons of OPFRs were consumed worldwide, whereas consumption reached 500,000, 680,000, and 1,050,000 tons in 2011, 2015, and 2018, respectively^1,32^. It has been reported that 56 OPFR monomers and 62 OPFR mixtures are currently produced in 367 factories around the world, with the top five OPFRs being triethyl phosphate (TEP) (~ 18%), tris (chloroisopropyl) phosphate (TCPP) (12%), TIBP, bisphenol-A bis (diphenyl phosphate) (BADP), and TCEP (9% each)^32,33^. Halogenated OPFRs, including TDCPP, TCEP, and TCPP, are mainly used in commercial products, such as furniture, textiles, mattresses, and electronics^32^. This promoted the entry and distribution of OPFRs in various environmental matrices. It has been shown that OPFRs can be ubiquitously detected, even in the Antarctic air, with up to 92.3 ± 13.8 pg/m^3^^34^. The parent and metabolites of the OPFRs were also quantified in the blood and urinary samples of the populations. Ma et al. measured six OPFRs (TCEP, TPHP, o-TCrP, m-TCrP, and p-TCrP) in the serum of residents in Shandong, China, and found that the mean concentrations of the total six OPFRs increased from 680 ng/g lipid in 2011 to 709 ng/g lipid in 2015, and TCEP was the most abundant, contributing a mean of 82% of the total 6 OPFRs concentrations^35^. It was also shown that the concentrations of OPFRs, including TBP, TBEP, TCEP, TCPP, TPHP, and tris (2-ethylhexyl) phosphate (TEHP), in the plasma of citizens in Zhejiang, China, ranged from 1.191 to 13.030 ng/mL. TPHP, TBEP, and TBP have the highest detection frequencies (95–100%) in plasma and blood cells^26^. The California Household Exposure Study detected TCEP and its metabolites in 13% and > 75% of assayed urinary samples, respectively^36^. In the present study, TBP, TBEP, TCEP, TDCPP, TMCP, and TOCP were detected in more than 90.0% of the urinary samples, suggesting exposure to OPFRs in the studied populations.

Environmental exposure to OPFRs in populations mainly occurs through inhalation, digestion, and dermal contact^37^. Upon absorption, OPFRs are primarily metabolized into organophosphate diesters and hydroxylated products in the human body. Therefore, their metabolites in urine have been proposed as biomarkers of internal exposure to OPFRs^38^. However, the urinary metabolites of OPFRs (TCEP, TDCPP, and TPHP) were weakly correlated with external exposure (house dust)^36,39^. Additionally, a metabolite may be derived from different parent OPFR compounds, such as diphenyl phosphate (DPHP), which is a potential metabolite of several phosphates containing at least two phenyl substituents, including TPHP and 2-ethylhexyl diphenyl phosphate (EHDPP)^36^. Moreover, because metabolic pathways and enzymes have not been fully elucidated, some metabolites of OPFRs, such as tris (2-butoxyethyl) phosphate (TBOEP), TCEP, TCPP, TDCPP, and TPHP, have not been identified in animal models or in vitro metabolic experiments^40–42^. Therefore, to obtain more accurate information on population exposure, both parent OPFRs and their metabolites (e.g., TCEP and BCEP) have been proposed for use in biomonitoring studies^42^. In the present study, we found that media concentrations of seven OPFRs in urine ranged from 1.444 to 23.616 ng/mg creatinine (1.208–17.668 ng/mL). The most predominant OPFR was TDCPP, followed by TBEP, TMCP, TOCP, TCEP, TPHP, and TBP, which seemed to be higher than those measured in the southern Taiwanese populations (TDCPP, 0.0214 ± 0.0632 ng/mL; TCEP, 0.682 ± 1.260 ng/mL; TBEP, 0.394 ± 0.776 ng/mL; TPHP, 0.0342 ± 0.110 ng/mL; and tri-n-butyl phosphate (TNBP) 0.0380 ± 0.0505 ng/mL)^43^. Environmental exposure to OPFRs has been reported to be significantly associated with individual behaviors such as the frequency of eating out and hand-washing habits before eating^43^. The high exposure to OPFRs in the present study is probably related to environmental pollution in Liuzhou, a heavy industrial city, and the living behaviors of residents whose health literacy levels are lower than those in other areas in China^44^. Further investigation of the exposure characteristics and the relationship between external and internal exposure to OPFRs in populations will provide more information.

Using OPFR mixtures and co-using OPFR monomers might lead to correlations between OPFRs and their metabolites in the urine. Dodson et al. measured the urinary metabolites of six OPFRs among California residents and showed that DPHP (TPHP metabolite) was correlated with BDCPP (TDCPP metabolite), BCEP (TCEP metabolite), and DBP (TBP metabolite), and that the latter was correlated with BCEP and BDCPP^36^. For newborns in Southern Taiwan, there was a correlation between urinary TDCPP and TPHP, as well as between TNBP and TPHP, whereas for the 1–17-year-old population, TNBP was correlated with TBEP and TDCPP^43^. Correlations between OPFRs suggest common exposure sources and provide information on cumulative exposure and health effects. In the present study, TBP was correlated with TCEP, TDCPP, TOCP, and TBEP, whereas TCEP was correlated with TBEP in the urine of the pregnant women. These correlations may also provide the basis for BKMR analysis and for designing mixed exposures to OPFRs in future studies.

### Association between OPFRs and GDM

To explore the association between exposure to OPFRs and GDM, WQS and BKMR models were used to estimate the effects of individual, mixture, and interaction of OPFRs on GDM risk in this study. The results showed that TBP and TPHP accounted for the majority of the WQS index weights. The BKMR model reinforced these findings, showing that joint exposure to OPFRs and individual exposure to TBP, TMCP, and TPHP or interactions of TMCP with TBP and TPHP were significantly associated with GDM. The median concentrations of serum TDCPP, TOCP, TNBP, TBOEP, TIBP, and TCIPP in GDM were significantly higher than those in the non-GDM group. However, only TBOEP (OR 2.63; 95% CI 1.68, 4.11), TNBP (OR 2.07; 95% CI 1.27, 3.41), and TPHP (OR 1.03; 95% CI 1.05, 1.51) were associated with GDM, in which the authors attributed to a smaller sample size of controls than the GDM group (130 GDM and 67 controls)^27^. Studies have shown that TPHP have reproductive, developmental, endocrine, and metabolic toxicity^45,46^. Morris et al. revealed that OPFRs may inhibit specific liver carboxylesterase activity in exposed mice and lead to changes in liver lipid metabolism, thereby causing diabetes^47^. Hu et al. found that exposure to TPHP might disturb the biosynthesis of progesterone in human placental villus cancer cells, implying that TPHP influenced female reproduction and fetal development^45^. In mice exposed to TPHP (orally 300 mg/kg for 35 days), malondialdehyde levels in the liver were significantly increased, while glutathione content was decreased. Furthermore, pathological injury in testicular tissue and a decrease in testosterone levels were observed. The expression of steroid-acute regulatory protein (StAR), low-density lipoprotein receptor (LDL-R), cytochrome P450 cholesterol side-chain cleavage enzyme (P450scc), and cytochrome P450 17α-hydroxysterol dehydrogenase (P450-17α) was reduced, indicating TPHP-induced oxidative stress and endocrine disorders in mice^46^. Studies have also shown that TPHP and TBP have antagonistic activities against human estrogen receptor α (Erα) and/or β (Erβ), androgen receptor (AR), glucocorticoid receptor (GR), and progesterone X receptor (PXR), supporting the effect of TBP and TPHP on endocrine disruption which may increase the risk of GDM development^48,49^.

The study reported a positive association between urinary OPFRs and GDM based on a nested case–control study, and this association was confirmed using different statistical models. However, all participants were South Chinese residents, and the results of the study may have some geographical limitations.

## Conclusion

Mixed exposure to seven OPFRs, including TBP, TBEP, TCEP, TDCPP, TMCP, TOCP, and TPHP; individual exposure to TBP, TPHP, and TMCP; and interactions of TMCP with TBP and TPHP, were significantly positively associated with GDM, indicating that OPFRs have adverse health effects on pregnant women.

## Data Availability

The datasets used and analyzed during the current study are available from the corresponding author on reasonable request.
